# Uneven distribution of prokaryote-derived horizontal gene transfer in fungi: a lifestyle-dependent phenomenon

**DOI:** 10.1128/mbio.02855-24

**Published:** 2024-11-29

**Authors:** Fei Liu, Shi-Hui Wang, Ratchadawan Cheewangkoon, Rui-Lin Zhao

**Affiliations:** 1 State Key Laboratory of Mycology, Institute of Microbiology, Chinese Academy of Sciences 85387 , Beijing, China; 2 Department of Entomology and Plant Pathology, Faculty of Agriculture, Chiang Mai University, Chiang Mai, Thailand; 3 Department of Biology, Faculty of Science, Chiang Mai University, Chiang Mai, Thailand; 4 College of Life Science, University of Chinese Academy of Sciences, Beijing, China; University of Cambridge, Cambridge, United Kingdom

**Keywords:** horizontal gene transfer, fungal genomes, gene acquisition, functional implications, lifestyle-related traits

## Abstract

Horizontal gene transfer (HGT) in fungi is less understood despite its significance in prokaryotes. In this study, we systematically searched for HGT events in 829 representative fungal genomes. Using a combination of sequence similarity and phylogeny-based approaches, we detected 20,093 prokaryotic-derived transferred genes across 750 fungal genomes, via 8,815 distinct HGT events. Notably, our analysis revealed that eight lifestyle-related traits significantly influence HGT diversity in fungi. For instance, parasites exhibited the highest estimated number of HGT-acquired genes, followed by saprotrophs, with symbionts showing the lowest. HGT-acquired genes were predominantly associated with metabolism and cellular functions and underwent purifying selection. Moreover, horizontally transferred genes with introns have significantly higher expression levels compared to intron-lacking genes, suggesting a probable role of intron gains in the adaptation of HGT-acquired genes. Overall, our findings highlight the influence of lifestyle on HGT diversity in fungi and underscore the substantial contribution of HGT to fungal adaptation.

**IMPORTANCE:**

This study sheds new light on the role of horizontal gene transfer (HGT) in fungi, an area that has remained relatively unexplored compared to its well-established prevalence in bacteria. By analyzing 829 fungal genomes, we identified over 20,000 genes that fungi acquired from prokaryotes, revealing the significant impact of HGT on fungal evolution. Our findings highlight that fungal lifestyle traits, such as being parasitic or saprotrophic, play a key role in determining the extent of HGT, with parasites showing the highest gene acquisition rates. We also uncovered unique patterns of HGT occurrence based on fungal morphology and reproduction. Importantly, genes with introns, which are more highly expressed, appear to play a crucial role in fungal adaptation. This research deepens our understanding of how HGT contributes to the metabolic diversity and ecological success of fungi, and it underscores the broader significance of gene transfer in shaping fungal evolution.

## INTRODUCTION

Horizontal gene transfer (HGT) is the sharing of genetic material between organisms that are not in a parent-offspring relationship (1). While HGT is a well-documented phenomenon in prokaryotes, where it is recognized as a critical mechanism for genomic innovation and adaptation in bacteria and archaea, its study in eukaryotes, particularly complex multicellular forms, has lagged behind (2, 3). The frequency, mechanisms, and impact of HGT between bacteria and eukaryotes remain subjects of active debate and investigation, with many questions still unresolved (4–6).

In eukaryotes, the mechanisms facilitating HGT are less understood compared to prokaryotes, where horizontal transfers are often facilitated by plasmids, phages, or other mobile genetic elements (7, 8). Recent discoveries of giant transposable elements in fungi, which harbor numerous genes associated with HGT events, highlight the presence and potential importance of these genetic exchanges within the fungal kingdom (8, 9). These findings suggest that HGT might play a significant role in fungal evolution, although the precise mechanisms and implications are not yet fully elucidated.

Fungi are ecologically versatile organisms that act as plant pathogens, symbionts, and decomposers, making them an excellent model for studying HGT in complex multicellular eukaryotes (10). The availability of extensive genomic data from a wide range of fungal species, including both unicellular and multicellular forms, provides a unique opportunity to explore HGT’s role in fungal evolution and adaptation (10). Previous studies have demonstrated the presence of prokaryotic genes in fungi, often focusing on specific species or groups (10–17). For example, the acquisition of bacterial genes by HGT in plant-associated fungi is widespread and considered a significant driving force in their adaptive evolution, with these genes encoding factors crucial for niche specification, pathogenicity, and adaptation to diverse metabolic requirements (18, 19). Phylogenetic studies have revealed that bacterial proline racemases have been transferred into fungal genomes (15), and several fungal species from the *Colletotrichum* genus possess bacterial genes (16). Moreover, the transfer of pore-forming toxins from bacteria to eukaryotes has been observed, indicating multiple independent HGT events involving these toxin genes (20).

Despite these insights, the role of prokaryote-derived HGT in fungal evolution remains poorly understood. For example, a study by Shen et al. (21) examined HGT in 332 yeast genomes and identified 878 putative HGT-acquired genes, suggesting that while HGT occurs, its prevalence may be lower than expected. The fate of foreign genes after integration into host genomes is influenced by factors such as gene length, structure, and the surrounding genomic environment (4). Notably, intron gains have been shown to enhance the retention of HGT-acquired genes by increasing their expression, particularly when these genes have functional roles (4, 22).

Despite some progress, the role of prokaryote-derived HGT in fungal evolution remains an enigma. Understanding the overall extent and pattern of HGT events in fungi, as well as their precise impact on complex multicellular fungal evolution, remains uncertain. While various selfish genetic elements like mycoviruses (23) and transposable elements (9) have been noted for horizontal transfer, the mechanisms of HGT in fungi remain incompletely elucidated. To address these gaps, we conducted a comprehensive investigation into prokaryote-derived HGT present in fungal genomes, with a particular focus on complex multicellular Dikarya, the most species-rich fungal group. Dikarya encompasses lineages with diverse ecological roles and levels of morphological complexity, making it an ideal model for understanding HGT in fungi. Notably, mushroom-forming fungi (Agaricomycetes) exhibit the greatest morphological diversity and complexity within this group, positioning them as a particularly intriguing focus for exploring the dynamics of HGT in complex multicellular organisms.

Our study covered fungi exhibiting diverse lifestyle-related traits, ranging from unicellular to complex multicellular forms, and from saprotrophs to parasites and symbionts. Our aim was to elucidate the functions and contributions of HGT to fungal adaptation, including an exploration of the potential origins, functions, selection pressure, and adaptation mechanisms of the genes acquired through HGT in fungi. Through this research, we seek to deepen the understanding of the role of HGT in fungal evolution and ecological adaptation, particularly its association with fungal lifestyle. This study sheds light on how fungi maintain their diversity and functionality within complex ecosystems through genetic exchange.

## RESULTS

### Horizontal gene transfer detection in fungal genomes

**Fig 1:**
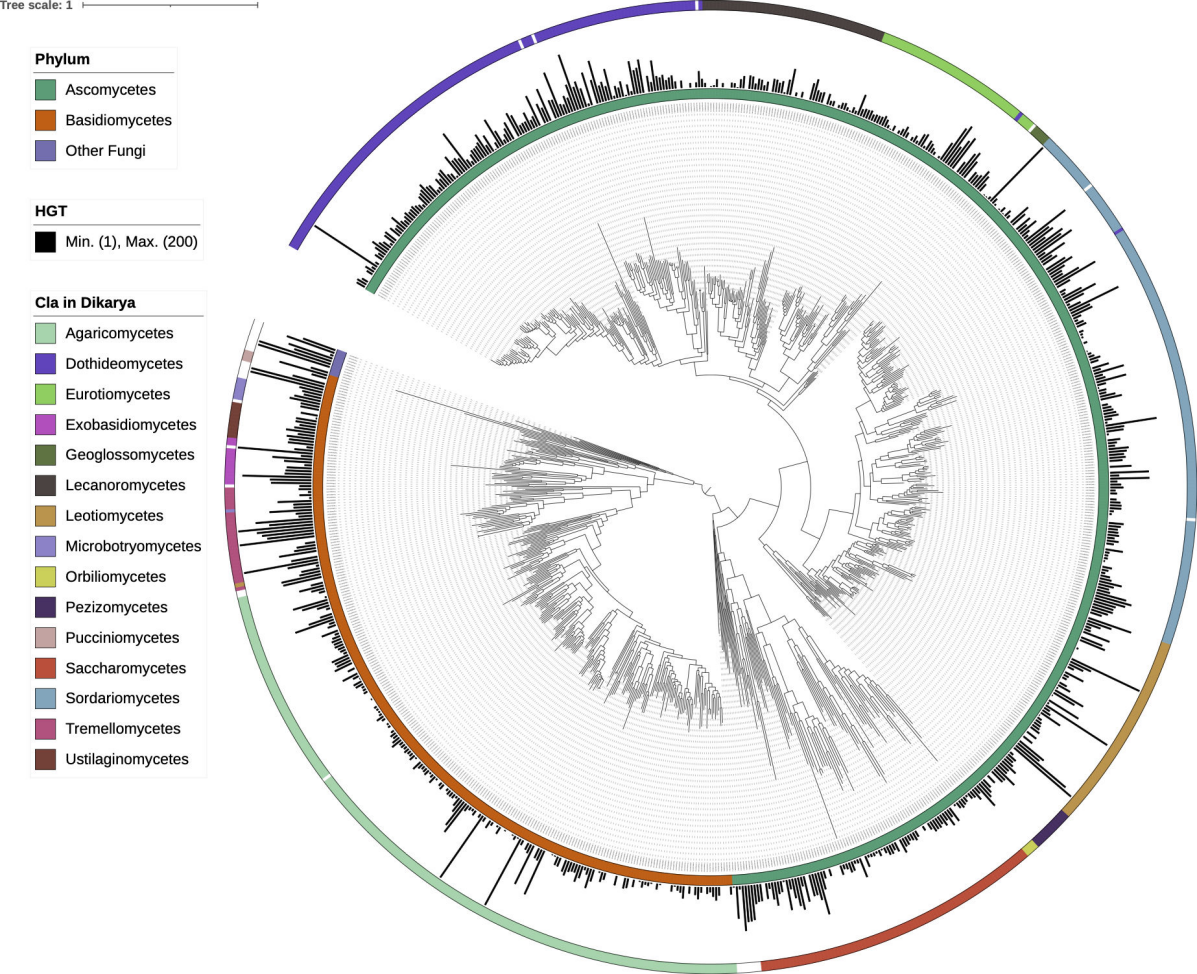
Distribution of putative HGT-acquired genes on phylogeny. This figure depicts the distribution of 20,093 putative HGT-acquired genes across a maximum likelihood phylogeny of 829 fungal genomes. The phylogeny is based on a concatenated maximum likelihood tree derived from the analysis of 758 single-copy, full-length BUSCO genes. Major taxonomic groups are represented by rectangles in the outer and inner rings, denoting phyla and classes, respectively. Bars indicate the number of HGT-acquired genes per genome.

To systematically identify putative HGT-acquired genes in fungi, specifically within complex multicellular Dikarya, we downloaded 829 species representative genomes from GenBank (see Materials and Methods). These genomes represent two major phyla, primarily comprising the Ascomycota (563 genomes) and Basidiomycota (257 genomes) phyla. Among these genomes, there are representatives from 11 classes with at least 10 genomes each (Fig. 1), including Agaricomycetes (188), Sordariomycetes (151), Dothideomycetes (139), Saccharomycetes (82), Leotiomycetes (57), Lecanoromycetes (52), Eurotiomycetes (48), Tremellomycetes (27), Pezizomycetes (12), Exobasidiomycetes (12), Ustilaginomycetes (10). We identified a total of 9,597,840 genes from 829 fungal genomes, with 7,091,363 of these genes present in contigs longer than 100 kb. Using sequence similarity and phylogeny-based approaches, we examined the protein sequences of these 7,091,363 genes for evidence of HGT. Initially, 29,796 candidate HGT genes were identified. However, 9,703 of these did not pass phylogenetic validation, resulting in a final set of 20,093 putative HGT genes. The Newick files for the phylogenetic trees of these genes are provided in the supplementary data sets.

Our analysis revealed that 20,093 genes across 750 fungal genomes were likely acquired from prokaryotes and viruses (Fig. 1; Table S1). Of these, 19,973 (99.4%) were from bacteria, 78 (0.4%) from archaea, and 42 (0.2%) from viruses. The phylogenies of these HGT-acquired genes indicate they stem from 8,815 distinct HGT events: 6,953 were species-specific, and 1,862 involved two or more species (Table S2). These horizontally transferred genes constitute 0.21% of the total genes analyzed.

Additionally, to illustrate the relationship between HGT frequency and overall gene content, we performed a Pearson’s correlation analysis, comparing the number of HGT-acquired genes to the total gene content across our data set (Fig. S1). Notably, we found no correlation between the number of HGTs and the total number of genes in a fungal genome (*P*-value = 0.18), suggesting that genome size does not influence the incorporation of transferred genes. This finding indicates that HGT is a widespread phenomenon in fungi, regardless of their genome size.

### Validation of horizontally transferred genes

**Fig 2:**
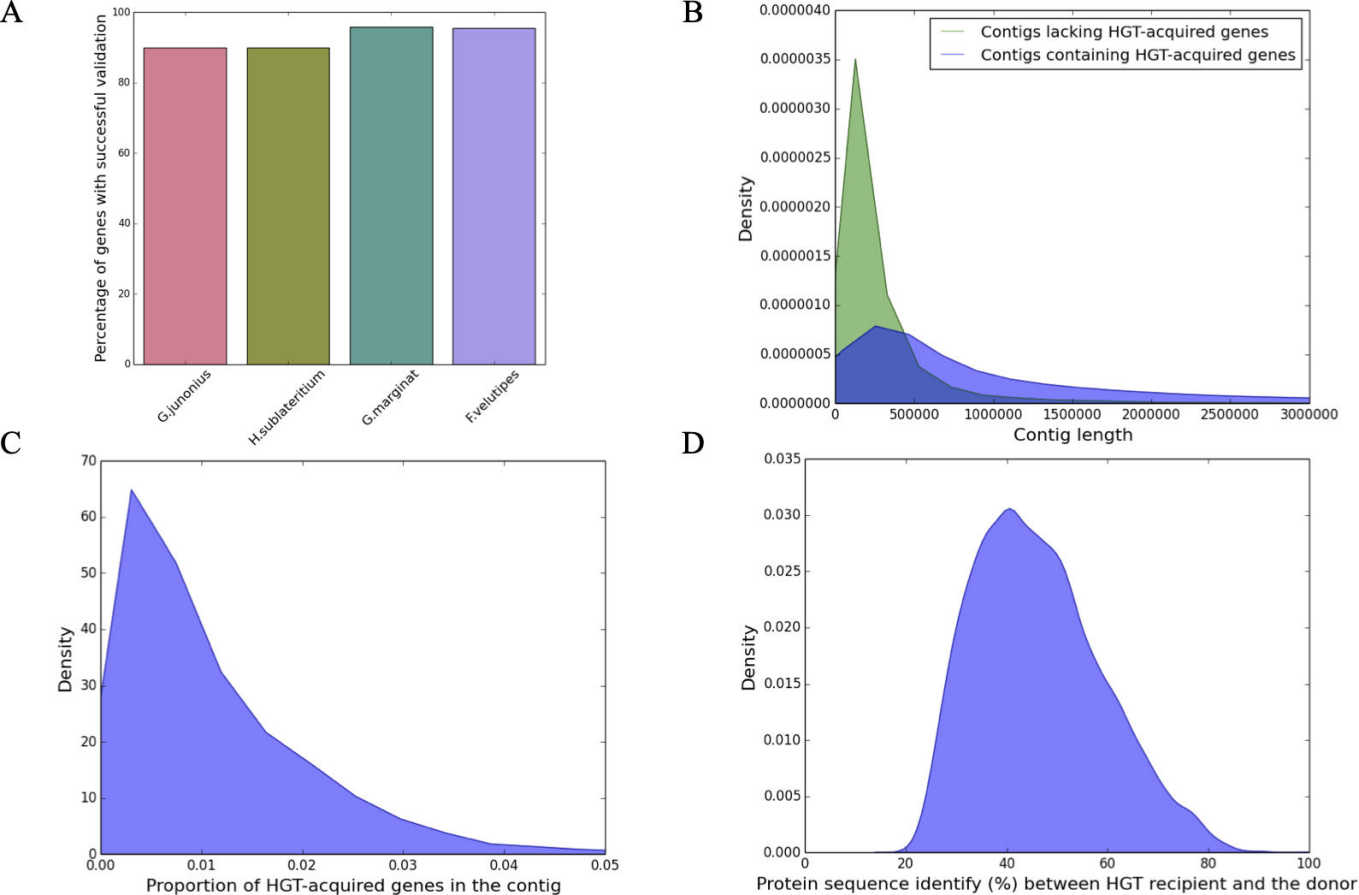
Robustness of HGT inference. (**A**) Validation of HGT-acquired genes using PacBio sequencing. To address the challenge of validating all HGT-acquired genes in fungi, 66 HGT-acquired genes were examined across four fungal species (*Gymnopilus junonius*, *Hypholoma sublateritium*, *Galerina marginat*a, *and Flammulina velutipes*). (**B**) Distributions of sequence lengths of genomic contigs with (blue) and without (green) HGT-acquired genes. (**C**) Distribution of proportions of HGT-acquired genes in each of the 9,794 contigs that harbor the 20,093 inferred HGT-acquired genes. (**D**) Distribution of protein sequence similarity between the sequence in the fungal recipient genomes and their closest donor genome hits for all HGT-acquired genes.

To ensure the 20,093 HGT-acquired genes were not contaminants, we used Conterminator v1.c74b5. We then calculated the recovery rate of 66 randomly chosen genes from four fungi, with results between 90.0% and 95.8%, averaging 94.0% (62/66 genes) using PacBio/Illumina sequencing (Fig. 2A). Comparison with previously identified HGT-acquired genes in budding yeast (21) revealed a high degree of overlap with our findings. By applying identity thresholds of 50%, 60%, 70%, 80%, 90%, 95%, and 99%, we observed that 80%–91% of these genes were also identified in our study. This overlap proportion, achieved by excluding HGT genes not present in our original protein sequence data set, indicates a higher degree of consistency when directly comparable sequences are considered. An examination of the distribution of sequence lengths of the 9,794 genomic contigs containing the HGT-acquired genes alongside those of the 36,507 genomic contigs without HGT-acquired genes, we found that contigs containing the HGT-acquired genes were typically longer than those lacking them (Fig. 2B). We calculated the proportion of HGT-acquired genes relative to the total gene count across 9,794 contigs, finding it to be as low as 1.1% (Fig. 2C). The distribution of proportions of HGT-acquired genes residing in the 9,794 contigs was as low as 1.1%. Finally, we examined the protein sequence similarity between the HGT-acquired genes in the fungal recipients and their closest homologs in non-fungal best hits for all 20,093 HGT-acquired genes and found that the average identity value was 46% (Fig. 2D). To provide a baseline for expected percentage identity values for vertically inherited genes, we included the distribution of hits for these non-HGT genes. We then used the Wilcoxon rank-sum test to compare the percentage identity distributions of HGT candidate genes and non-HGT genes. The results indicated a significant difference between the two distributions (*P*-value < 0.05). These results support the reliability of our list of 20,093 HGT-acquired genes.

### HGT distribution and the impact of lifestyle-related traits on HGT diversity in fungi

**Fig 3:**
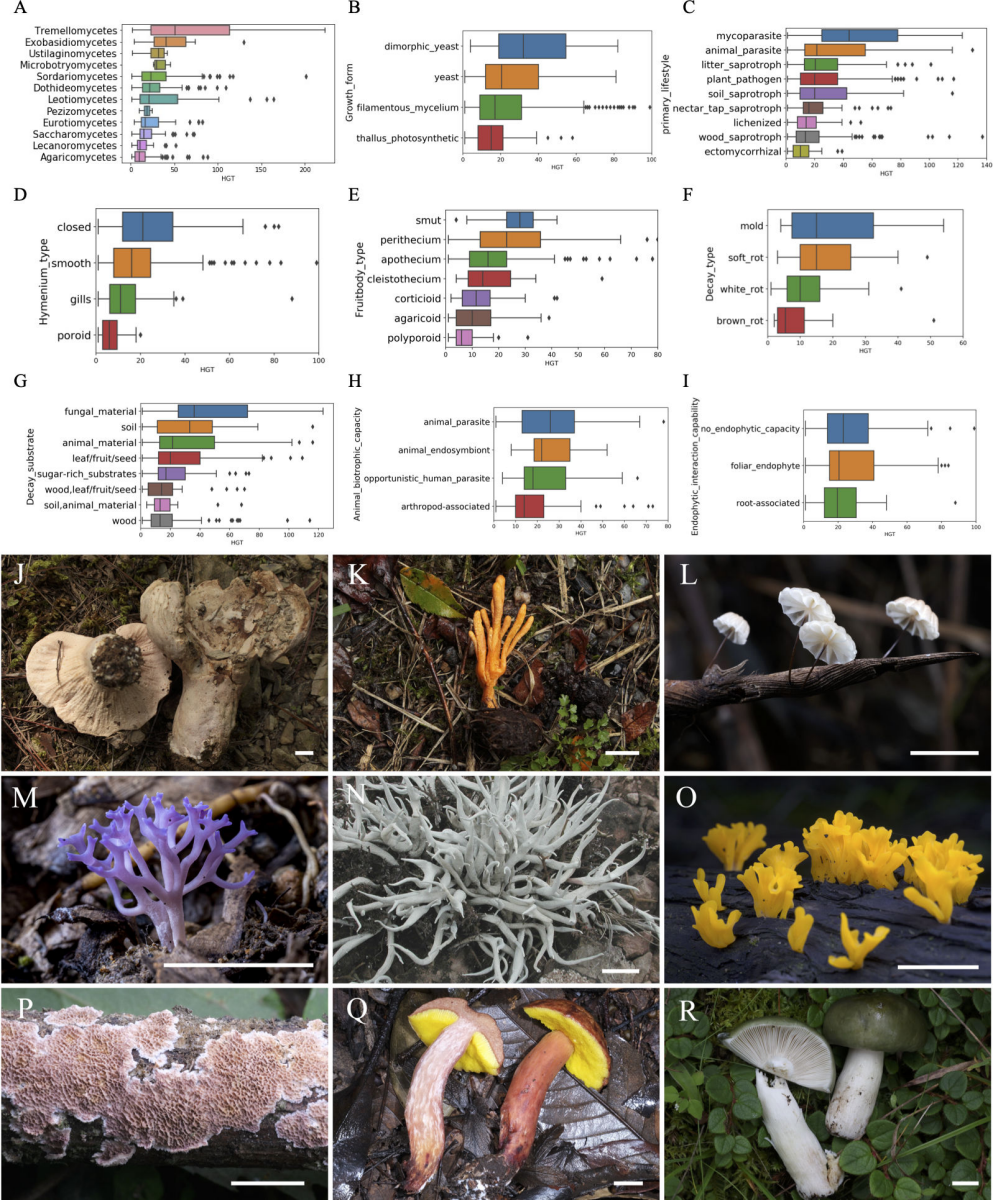
HGT distribution in different fungal groups. This box plot illustrates the number of HGT-acquired genes across various categories: dominant classes (**A**): Tremellomycetes (47 HGT-acquired genes) and Exobasidiomycetes (38) have the highest median number of HGT-acquired genes, followed by Ustilaginomycetes (36), Microbotryomycetes (30), Pezizomycetes (23), Sordariomycetes (20), Dothideomycetes (19), Leotiomycetes (17), Eurotiomycetes (16), Saccharomycetes (15), and Lecanoromycetes (13). Agaricomycetes demonstrate the lowest median number of HGT-acquired genes (10). Primary lifestyle (**B**): categories include plant pathogen (169 genomes), wood saprotroph (160), soil saprotroph (72), litter saprotroph (60), nectar/tap saprotroph (52), lichenized (47), animal parasite (42), and ectomycorrhizal (29). Decay type (**C**): six categories exist, with white rot (74), soft rot (28), brown rot (18), and mold (10) being the most common. Mold exhibits the highest estimated number of HGT-acquired genes, followed by soft rot and white rot, with brown rot showing the lowest. Decay substrate (**D**): over 40 categories exist, with wood (164), leaf/fruit/seed (105), sugar-rich substrates (52), and soil (48) being prevalent. Fungal material and soil exhibit the highest estimated number of HGT-acquired genes, while wood shows the lowest. Animal biotrophic capacity (**E**): categories include arthropod-associated (63), opportunistic human parasite (33), animal parasite (21), and animal endosymbiont (11). Plant pathogenic capacity (**F**): leaf/fruit/seed pathogen (151), wood pathogen (44), and root-associated (15) are common categories. Growth form (**G**): genomes are classified into filamentous mycelium (526), yeast (112), thallus-like photosynthetic (48), and dimorphic yeast (43). Hymenium type (**H**): perithecium (157), apothecium (81), agaricoid (61), corticioid (39), polyporoid (37), cleistothecium (15), smut (12), and clavarioid (10) are prevalent. Smut and perithecium exhibit the highest estimated number of HGT-acquired genes, while polyporoid and agaricoid show the lowest. Fruitbody type (**I**): closed (194), smooth (123), gills (54), and poroid (47) are prevalent. Closed fruitbody type exhibits the highest estimated number of HGT-acquired genes, while poroid shows the lowest. Examples of fungi with different lifestyles (J–R): J, *Hypomyces lactifluorum*, showing a mycoparasitic lifestyle. K, *Cordyceps militaris*, displaying animal parasitism. L, *Marasmius epiphyllus*, functioning as a litter saprotroph. M, *Ramariopsis pulchella*, serving as a soil saprotroph. N, *Thamnolia vermicularis*, showing a lichenized form. O, *Dacryopinax spathularia*, acting as a wood saprotroph. P, *Trichaptum abietinum*, representing a corticioid form. Q, Hymenium of *Aureoboletus zangii*, exhibiting polyporoid and ectomycorrhizal characteristics. R, *Russula subalpinogrisea*, displaying ectomycorrhizal and agaricoid features. Scale bars = 10 mm.

Upon evaluating the HGT-acquired genes from each phylum, our findings reveal that the phylum Ascomycota exhibits a higher median number of HGT-acquired genes (16 genes per genome) compared to the phylum Basidiomycota (14). Furthermore, an assessment of the HGT-acquired genes from each class exposed that the classes Tremellomycetes and Exobasidiomycetes recorded the highest median number of HGT-acquired genes, followed by the class Ustilaginomycetes, Microbotryomycetes, Pezizomycetes, Sordariomycetes, Dothideomycetes, Leotiomycetes, Eurotiomycetes, Saccharomycetes, Lecanoromycetes, while Agaricomycetes had the lowest (Fig. 3A).

To assess the impact of lifestyle-related traits on HGT diversity, we annotated 17 such traits for each fungal genome using the FungalTraits database (Table S3) and quantified their relative influence on HGT diversity using redundancy analysis (RDA). Our analysis revealed that eight lifestyle-related traits, including primary lifestyle, decay type, decay substrate, animal biotrophic capacity, growth form, hymenium type, fruitbody type, and endophytic interaction capability, all showed significant effects (*P*-value < 0.05; Table S4). Additionally, to test whether HGT is associated with lifestyle accounting for phylogeny, we performed the phylogenetically corrected regression analysis using phylogenetic generalized least squares (PGLS): eight lifestyle-related traits all showed significant effects (*P*-value < 0.05; Table S4).

The most commonly occurring lifestyle is listed in the “primary_lifestyle” field, with 19 categories. Among these, plant pathogen, wood saprotroph, soil saprotroph, litter saprotroph, nectar/tap saprotroph, lichenized, animal parasite, and ectomycorrhizal were the most prevalent (Fig. 3B). Notably, mycoparasite and animal parasite exhibited the highest estimated number of HGT-acquired genes, followed by litter saprotroph, plant pathogen, and soil saprotroph, with lichenized, wood saprotroph, and ectomycorrhizal fungi displaying the lowest (Fig. 3B). The mycoparasite *Xylogone* sp. PMI 703 (GCA_021399445.1) was among the top 10 genomes with the highest number of HGT-acquired genes. Furthermore, when we categorized primary lifestyles into three groups: saprotrophs, parasites, and symbionts, parasites exhibited the highest estimated number of HGT-acquired genes, followed by saprotrophs, with symbionts showing the lowest.

Of saprotrophs, we emphasize two primary characteristics: decay type and decay substrate. Among decay types, white rot, soft rot, brown rot, and mold were the most prevalent. Interestingly, mold exhibited the highest estimated number of HGT-acquired genes, followed by soft rot and white rot, with brown rot showing the lowest (Fig. 3C). Regarding decay substrates, with over 40 categories, wood, leaf/fruit/seed, sugar-rich substrates, soil, animal material, wood/leaf/fruit/seed, fungal material, and soil/animal material were prominent. Notably, fungal material and soil exhibited the highest estimated number of HGT-acquired genes, while wood showed the lowest (Fig. 3D). One noteworthy finding was the mycoparasite *Xylogone* sp. PMI_703 (GCA_021399445.1), associated with fungal material decay type, ranks among the top 10 genomes with the highest number of HGT-acquired genes. Additionally, while aquatic habitat did not significantly affect HGT diversity in our RDA analysis, aquatic fungi exhibited a higher prevalence of HGT compared to their non-aquatic counterparts.

In our investigation of parasites, we examined two key traits: animal biotrophic capacity and plant pathogenic capacity. Animal biotrophic capacity encompassed arthropod-associated, opportunistic human parasites, animal parasites, and animal endosymbionts, with animal parasites demonstrating the highest estimated number of HGT-acquired genes and arthropod-associated displaying the lowest (Fig. 3E). Concerning plant pathogenic capacity, wood pathogens exhibited the lowest HGT counts (Fig. 3F). For instance, the plant pathogen *Calonectria ilicicola* (GCA_020809705.1), possessing leaf/fruit/seed pathogen capacity, was found to harbor 130 HGTs. In contrast, another plant pathogen, *Sparassis latifolia* (GCA_018466975.1), which primarily targets wood as its substrate, exhibited only 2 HGTs.

In our examination of symbionts, we focused on endophytic interaction capability. Notably, no endophytic capacity demonstrated a higher estimated number of HGT-acquired genes than those with endophytic capacity. For example, the fungus *Moniliella wahieum* (GCA_003971905.1), lacking endophytic capacity, was discovered to harbor 158 HGTs. In contrast, the root-associated endophytic fungus *Sabuloglossum arenarium* (GCA_022345705.1) exhibited only 1 HGT.

Finally, in our examination of morphology and reproductive structures, we considered three traits: growth form, hymenium type, and fruitbody type. Notably, there was a significant contrast in HGT counts between unicellular and multicellular fungi, with yeast-like fungi possessing the highest number of HGT-acquired genes, followed by filamentous fungi, while thallus-like photosynthetic fungi displayed the lowest average number of HGT-acquired genes (Fig. 3G). For instance, the yeast *Vanrija pseudolonga* (GCA_020906515.1) harbored the highest number of HGT-acquired genes (223) among both Basidiomycota and Ascomycota. Regarding hymenium type, smut and perithecium exhibited the highest estimated number of HGT-acquired genes, while polyporoid and agaricoid showed the lowest (Fig. 3H). Regarding fruitbody type, the closed fruitbody type exhibited the highest estimated number of HGT-acquired genes, while poroid showed the lowest (Fig. 3I). For instance, the plant pathogen *Calonectria ilicicola* (GCA_020809705.1), characterized by a perithecium hymenium type and closed fruitbody, ranked among the top 20 genomes with the highest number of HGT-acquired genes. Examples of fungi with different lifestyles are visually represented in Fig. 3J through R. These images illustrate characteristic fungal forms and lifestyles, further exemplifying the ecological diversity within the fungal kingdom.

### Origin, functional prediction, and purifying selection of horizontally transferred genes

**Fig 4:**
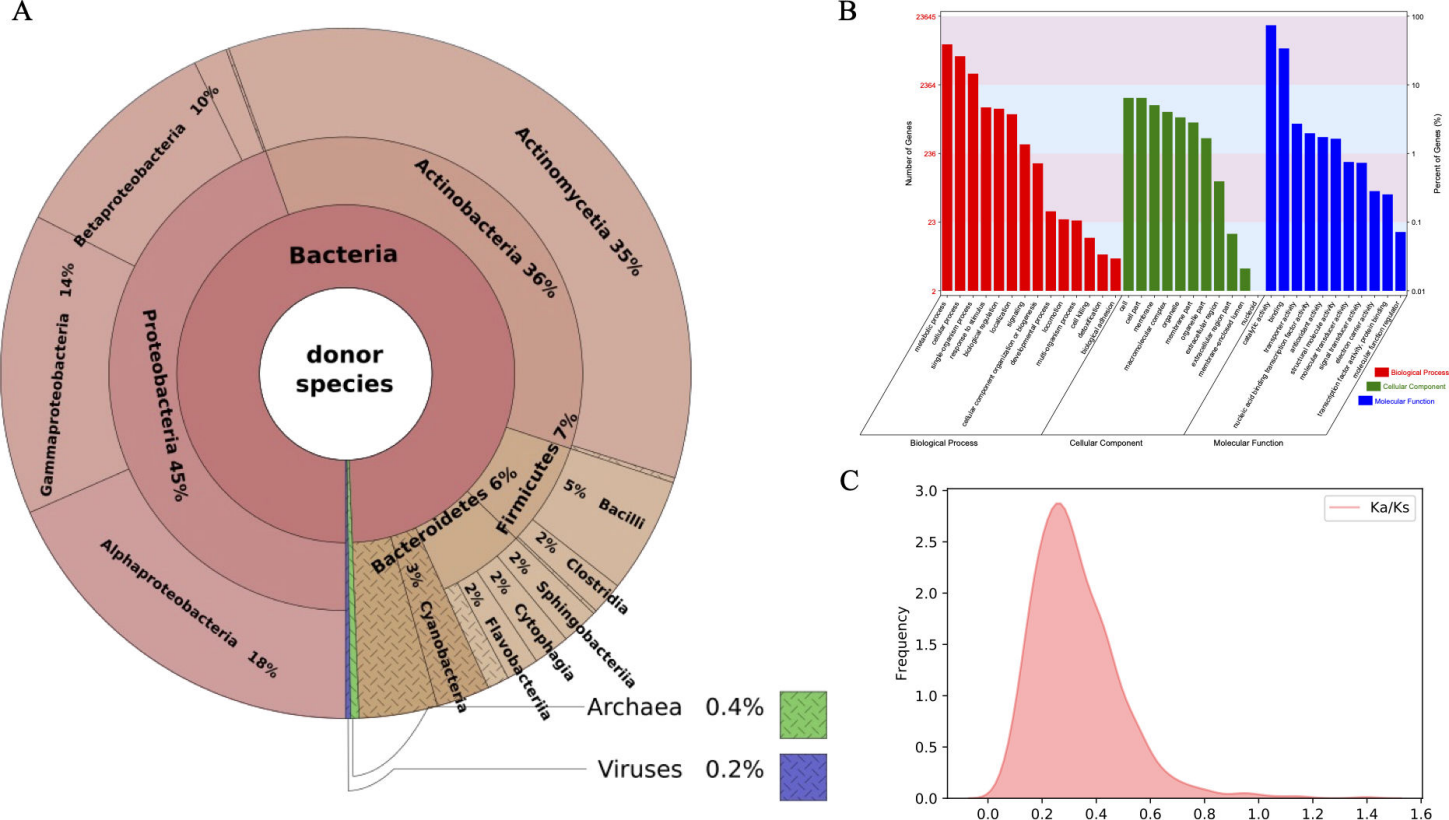
Origin, functional prediction, and selection of horizontally transferred genes. (**A**) Distribution of putative donor species for HGT-acquired genes. (**B**) Gene ontology (GO) term analysis of HGT-acquired genes categorized by biological process, cellular component, and molecular function. (**C**) Selection pressure of HGTs, shown as pairwise Ka/Ks ratios for horizontally transferred genes and their putative prokaryotic homologs.

Through an extensive analysis of putative donor organisms using BLAST and phylogenetics, we found that the majority of the identified 20,093 horizontally transferred genes in fungi were acquired from bacteria (99.4%). The donor bacterial groups identified were diverse, with Proteobacteria (45%), Actinobacteria (36%), Firmicutes (7%), and Bacteroidetes (6%) being the most prominent (Fig. 4A). The comprehensive examination provides a compelling picture of the diverse sources contributing to fungal genomes through HGT.

To explore the potential facilitation of gene transfer by transposable elements, we calculated the distance between every gene and its closest transposable element in each genome. We found that horizontally transferred genes were significantly closer to transposable elements (*P*-value = 2.03e-07). The functional prediction for each HGT-acquired gene with a potential donor is listed in Table S2. The top three annotated functions were short-chain dehydrogenases/reductases (SDR) family oxidoreductase, alpha/beta hydrolase, and GNAT family N-acetyltransferase. Gene ontology (GO) analysis of the 20,093 HGT-acquired genes demonstrated significant associations with metabolism- and cellular-related terms (Fig. 4B). Interestingly, this functional distribution remained consistent across the two phyla Ascomycota and Basidiomycota, affirming the robustness of our findings (Fig. S2).

Moreover, our study unveiled distinct GO term enrichments in HGT-acquired genes among fungi with different primary lifestyles, including response to stimulus, biological regulation, and protein folding (Fig. S3), highlighting the potential role of HGT in shaping functional adaptations within fungal populations. Notably, plant pathogens and wood saprotrophs emerged as the two primary lifestyles with the highest number of HGT-acquired genes, totaling 4,461 in plant pathogens and 3,344 in wood saprotrophs. Peroxidase activity and metabolic processes including superoxide were enriched in plant pathogens (Fig. S4), while transporter, signal transduction, and protein folding-related proteins were enriched in wood saprotrophs (Fig. S5). In the case of HGT-acquired genes among yeast, filamentous fungi, and thallus-like photosynthetic fungi, different enrichment GO terms were observed. The glutathione metabolic process was found to be enriched in yeast (Fig. S6), particularly in the class Tremellomycetes (Fig. S7), which includes yeasts and other unicellular fungi. Additionally, DNA-templated transcription, initiation, and signal transduction were enriched in filamentous fungi (Fig. S8), while translation was enriched in thallus-like photosynthetic fungi (Fig. S9).

Our analysis revealed several significant insights into the functional roles and potential applications of HGT-acquired genes. Kyoto Encyclopedia of Genes and Genomes annotations, in conjunction with GO results, highlighted key areas where horizontal gene transfer has impacted fungal metabolism and functions. Specifically, genes acquired through HGT were prominently associated with carbohydrate metabolism, amino acid metabolism, and xenobiotics biodegradation. Moreover, the identification of N-terminal secretion motifs in 1,938 genes and virulence factor annotations in 286 genes (260 from Pathogen Host Interactions [PHI] and 26 from Virulence Factor Database [VFDB]) underscores the potential role of HGT in fungal inter-species interactions and pathogenicity (Table S5). A notable example of HGT’s impact is observed in *Metarhizium robertsii*, which has emerged as a broad-host-range entomopathogen due to 18 specific HGT-acquired genes (24). Our study identified homologs of these genes in several species, including *Metarhizium brunneum* (Table S6). This finding suggests that similar HGT events might be contributing to the pathogenic capabilities of these species, potentially affecting their host range and virulence. Overall, these results emphasize the importance of HGT in shaping not only fungal metabolism and cellular functions but also their pathogenic potential.

Synonymous mutations typically result from neutral selection with no functional impact, while fixation of nonsynonymous mutations often indicates positive selection. In our analysis, we computed the number of nonsynonymous mutations (Ka), synonymous mutations (Ks), and their ratio (Ka/Ks) for HGT-acquired genes and their putative prokaryotic homologs. The majority of HGT genes exhibit Ka/Ks values below 0.5 (Fig. 4C), signifying negative selection. However, four genes—encoding alpha/beta fold hydrolase, GNAT family N-acetyltransferase, serine/threonine protein kinase, and HNH endonuclease—displayed Ka/Ks ratios greater than 1. This is often a sign of positive selection, suggesting these genes may have undergone adaptive evolution following horizontal transfer.

### Introns and adaptability of HGT in fungi

**Fig 5:**
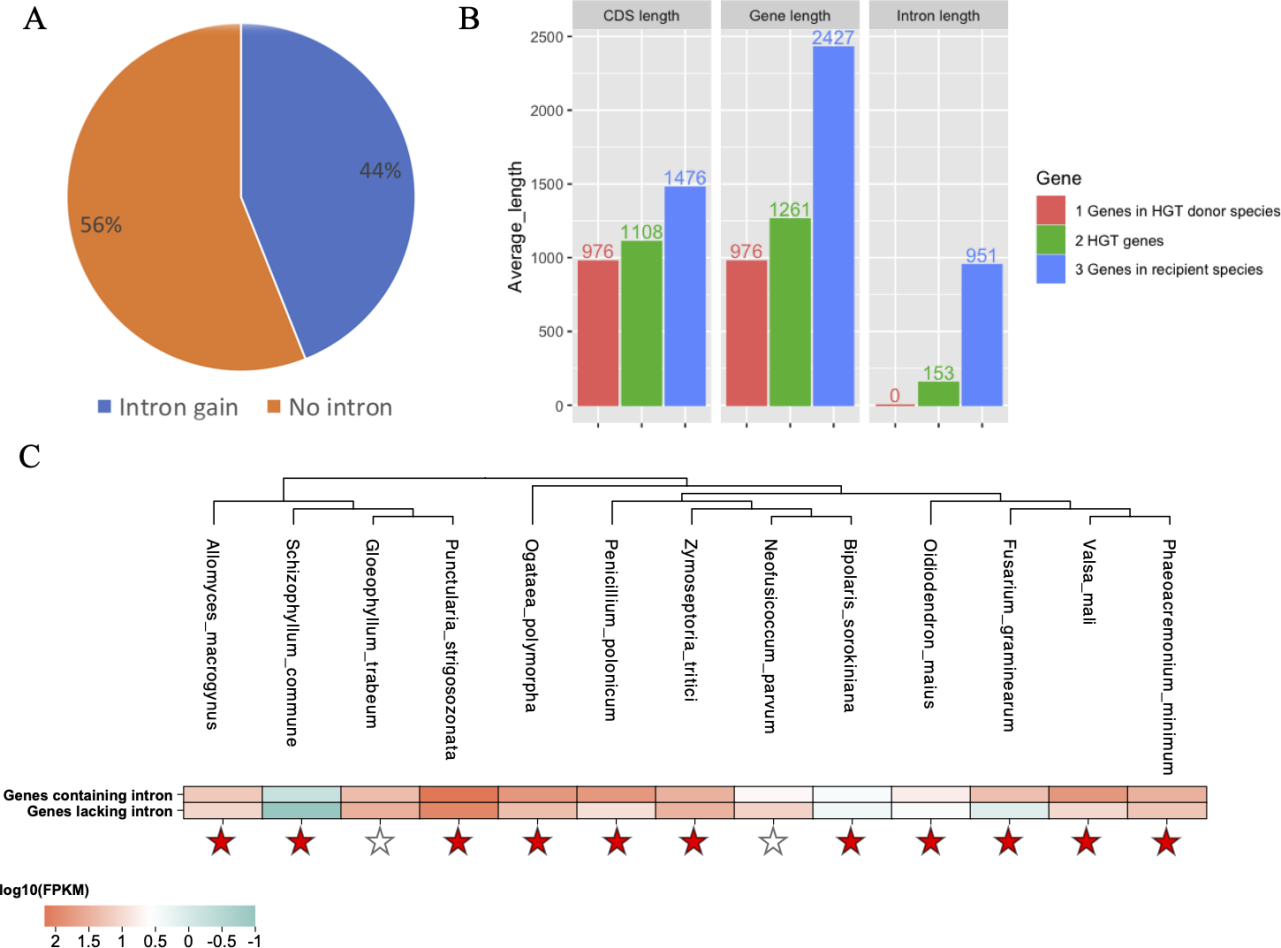
Intron gains from native fungal genomes and their role in HGT adaptation. (**A**) The proportion of HGT-acquired genes experiencing intron gains (blue) versus those without intron gains (orange) after integration into fungal genomes. (**B**) Comparisons of characteristics between transferred genes in HGT donor species (red), foreign genes in recipient species (green), and native genes in recipient species (blue), focusing on coding sequence length, gene length, and intron length. (**C**) Comparison of average expression level between HGT-acquired genes containing introns (I: first row) and without introns (II: second row) across 13 fungi. Expression levels are based on publicly available transcriptome data sets. The phylogeny of the 13 fungi was taken from the full phylogeny in Fig. 1. A red star indicates that the average expression level of HGT-acquired genes containing introns is higher than that of HGT-acquired genes lacking introns, while a white star indicates the opposite.

Analyzing the gene structure using GeneMark-ES, we observed that 44% of the horizontally transferred genes acquired introns, while 56% did not undergo any gain in introns following integration into fungal genomes (Fig. 5A). All 25,467 introns present in the genes were gained after these genes were inserted into fungal genomes. To identify the origins of these gained introns in the HGT-acquired genes, we carried out BLASTN searches of DNA sequences of introns against their native fungal genomes, with the option “-task blastn-short.” We found that 5,831/25,467 (23%) introns had BLAST hits (coverage >50%), with an average identity of 86%, whereas 19,636/25,467 (77%) had no BLAST hits (Fig. 5B).

Through an analysis of gene structures in HGT-acquired genes within putative donor and recipient genomes, as well as native fungal genes, we discovered that HGT-acquired genes in fungi had longer lengths than those of their counterparts in HGT donor species but were substantially shorter in length than native fungal genes (with an average length of acquired genes in recipient genomes at 1.3 kb, transferred genes in HGT donor genomes at 1.0 kb, and native genes in recipient genomes at 2.4 kb) (Fig. 5B). Comparatively, the coding sequence lengths of HGT-acquired genes in the putative donor and recipient genomes were similar to those of all native fungal genes (with an average length of foreign genes in recipient genomes at 1.1 kb, transferred genes in HGT donor genomes at 1.0 kb, and native genes in recipient genomes at 1.5 kb) (Fig. 5B). However, the length of introns in the HGT-acquired genes in the recipient genomes was longer than those in the putative donor genomes, yet shorter than those of native genes (with an average length of foreign genes in recipient genomes at 0.15 kb, transferred genes in HGT donor genomes at 0.09 kb, and native genes in recipient genomes at 0.95 kb) (Fig. 5B). This trend can be explained by intron gain events.

To investigate the potential role of intron gains in the adaptation of HGT-acquired genes to fungal genomes, we assessed the expression levels of HGT-acquired genes with and without introns across transcriptome data sets from each of the 13 fungi (Fig. 5C; Table S7). It’s important to note that this comparison was conducted exclusively within each transcriptomic data set, using data from the same developmental stage and tissue for a given species. Among the 13 fungal data sets, 11 (84.6%) exhibited higher expression levels for HGT-acquired genes containing introns as opposed to those lacking introns.

## DISCUSSION

### Distribution of HGT-acquired genes across fungi

Although previous efforts (11–13, 25, 26) have been significant in establishing the occurrence and ecological importance of HGT in fungi, a comprehensive understanding of HGT-acquired genes across the fungal kingdom remains incomplete. In our study, we conducted a comprehensive analysis to identify HGTs in fungal genomes and identified a total of 20,093 HGTs, representing approximately 0.21% of the total genes analyzed. This finding is consistent with previous research reporting that HGTs affect approximately 0.1% to 2.8% of the genes in a typical fungal genome (10, 11). The lack of correlation between the number of HGTs and proteome size is a particularly novel finding, suggesting that the overall amount of genetic material does not limit the incorporation of transferred genes. This broad distribution of HGTs across fungal genomes underscores the widespread nature of this phenomenon in the fungal kingdom.

### Impact of lifestyle-related traits on the diversity of HGT events

Our findings highlight that the lifestyle of fungi significantly impacts the number and diversity of HGT events. Parasitic fungi, which engage in dynamic interactions with their hosts, exhibited the highest number of HGTs. These interactions likely facilitate gene exchange, enhancing the parasite’s ability to exploit host resources. Saprotrophic fungi, which decompose a variety of substrates, also show a significant number of HGTs, reflecting their exposure to diverse microbial communities. Symbiotic fungi, which maintain stable relationships with their partners, exhibited fewer HGTs, suggesting a reduced reliance on external genetic material.

The substrate and decay type also play crucial roles in HGT occurrences. Soil, with its complex and diverse environment, promotes HGT due to its high microbial diversity (27, 28). Fungi that decompose a broad range of substrates, such as white and soft rot fungi, encounter more microbial communities and genetic material, leading to more HGTs compared to brown rot fungi, which specialize in narrower substrates. Also, the capacity for animal biotrophy in fungi can impact HGT, as different relationships and animal groups may facilitate or prevent gene transfer. Additionally, aquatic fungi exhibited a higher prevalence of HGT, possibly due to the elevated microbial diversity and density in aquatic habitats, facilitating gene transfer (29). HGT was more prevalent in freshwater fungi than in marine fungi, potentially due to the greater bacterial diversity in freshwater environments (30).

Morphological and reproductive traits of fungi also appear to influence HGT dynamics. Unicellular fungi exhibit higher HGT rates than multicellular fungi, likely due to the absence of a sequestered germ line and more frequent asexual reproduction (3, 31). For example, Tremellomycetes are a class of fungi that includes yeasts and other unicellular fungi, exhibiting a notable prevalence of HGT genes. Thallus-like photosynthetic fungi, which have a simpler evolutionary history arising from endosymbiosis with a green alga, likely have fewer opportunities for HGT due to their autotrophic lifestyle. Furthermore, different fruitbody types create varied ecological niches, affecting the types of microorganisms and genetic material fungi encounter, thus influencing the likelihood of HGT. Certain types of fruitbodies may produce compounds or structures that facilitate or inhibit the colonization of other microbes, such as bacteria or viruses, which can serve as vehicles for gene transfer. For instance, the class Agaricomycetes, known for their complex fruitingbodies, demonstrated the lowest median number of HGT-acquired genes.

### Origin and functions of HGT-acquired genes in fungi

Bacteria, particularly Proteobacteria, the largest phylum within the bacterial domain, were identified as the most frequent donors of HGT-acquired genes in fungi. This finding underscores the significant role of bacteria in shaping fungal genome evolution through HGT. Previous studies have discussed potential mechanisms of bacterium-to-eukaryote HGT, such as type IV secretion systems and bacteriophages as HGT vectors (5). Additionally, it has been proposed that the transfer of transposable elements could facilitate gene transfer (32, 33). Our study adds to this understanding by highlighting that horizontally transferred genes exhibit significant proximity to transposable elements, suggesting a strong association between transposable elements and HGTs in fungi.

Our functional analysis of HGT-acquired genes reveals a dominant functional classification covering a wide diversity of metabolic functions, suggesting HGT’s significant role in expanding and reconfiguring the core metabolic networks in fungi, consistent with previous studies (34). Many HGTs were predicted to encode N-terminal secretion motifs and participate in transportation, contributing to the enrichment of the fungal secretome and transporter repertoire. These transfers potentially enhance osmotrophic functions in numerous fungi (34). Furthermore, our study highlights a notable enrichment in transporter-related genes among wood saprotrophs, suggesting HGT’s involvement in shaping the protein repertoire essential for osmotrophy in fungi which aligns with a prior study (35).

Additionally, some HGT-acquired genes are related to virulence, potentially influencing the evolution of host adaptation in eukaryotic pathogens (36). For instance, Exobasidiomycetes, known for including plant pathogens like rusts and smuts, exhibited a notable prevalence of HGT genes, suggesting a potential link between pathogenicity and HGT acquisition. Specific examples include the chitinase gene *Bbchit1,* which can enhance the virulence of *Beauveria bassiana* for aphids (37), and was also found in the grapevine pathogen *Eutypa lata*, which is responsible for economic losses to the wine industry (38). Nine catabolic genes (AcdS, 1-aminocyclopropane-1-carboxylate deaminase) originating from Actinobacteria and Betaproteobacteria were found in our analyzed plant-related fungi, including *Calcarisporium arbuscula* and *Corinectria fuckeliana,* which aligns with a prior study (39). Moreover, a previous study experimentally verified the horizontal transfer of genes encoding bacterial lysozymes (cell wall-degrading enzymes) with potent antibacterial properties across diverse branches of the tree of life (40). In our study, we identified 20 lysozyme genes acquired via HGT in several fungal species (Table S2). The discovery of these lysozyme genes within the HGT-acquired gene pool opens up exciting possibilities for novel antibacterial strategies. Given their role in inhibiting bacterial growth, these genes could serve as a promising foundation for the development of new antimicrobial agents or therapeutic approaches targeting bacterial pathogens. Notably, HGT-acquired genes in plant pathogens may involve enzymes that either neutralize or utilize peroxidases and superoxide dismutases (Fig. S4), contributing to the pathogen’s survival, virulence, or interaction with the host plant (41, 42).

Furthermore, distinct GO term enrichments in HGT-acquired genes among fungi with different lifestyle-related traits, including response to stimulus, signal transduction, and protein folding. In our study, the set of 20,093 HGT-acquired genes includes both previously reported cases and discoveries spanning diverse functions such as metabolism, osmotrophy, and virulence. Our study suggests that HGT has played a pivotal role in the evolution of fungi, enabling adaptation to diverse ecological niches and providing insights into the complex interactions shaping fungal diversity and pathogenicity.

### Selection and adaptation of foreign genes in fungi

Selection is widely acknowledged as a major force influencing HGTs in bacteria, but ongoing debate exists regarding whether these transfers are generally beneficial, neutral, or possibly deleterious to the recipients (43). In the context of evolutionary selection, Ka/Ks values below 1 indicate negative selection, signifying selective pressure on gene sequences. Synonymous mutations, particularly in the third codon position, often fine-tune nucleotide composition without affecting function. In our study, we observed that low Ka/Ks values for horizontally transferred genes imply purifying selection, supporting their functionality (44). This finding is consistent with previous studies in plants (45).

Moreover, our analysis revealed that 44% of the acquired genes contained introns, likely obtained from native fungal genomes after the initial gene transfers (Fig. 5A). This is comparable to a previous study, which found that 40% of the acquired genes contained introns (10). Notably, the introns identified through BLAST analysis had an average sequence identity of 86%, while the horizontally transferred genes themselves showed an average identity of 46% with the donor sequences. This higher similarity in introns suggests they may have been acquired more recently, potentially indicating multiple rounds of HGT from closely related species. HGT-acquired genes with introns demonstrated significantly higher expression levels than those lacking introns (Fig. 5C). This finding is consistent with previous studies conducted across various organisms, including plants and insects, where intron gains were shown to enhance gene expression levels (22, 46, 47). Introns might enhance gene expression by affecting mRNA stability, splicing efficiency, and nuclear export (48–50). In summary, our findings suggest that introns acquired from native fungal genomes likely played a role in the adaptation of HGTs to recipient genomes.

In conclusion, our comprehensive analysis of 20,093 HGT-acquired genes across 829 fungal genomes offers new insights into the prevalence, origins, functions, and adaptive significance of HGT in fungi. The extensive presence of HGT-acquired genes illustrates the importance of these genes in enhancing fungal metabolic capabilities and environmental adaptability. Our study also underscores the significant impact of lifestyle-related traits on the occurrence and diversity of HGT events in fungi. The association between lifestyle traits and HGT diversity highlights how ecological interactions drive the incorporation of foreign genes. These findings reveal the widespread nature of HGT within the fungal kingdom and its pivotal role in shaping fungal genome evolution. Future research should delve deeper into the mechanisms facilitating HGT in fungi, the functional integration of these foreign genes into fungal genomes, and their specific impacts on fungal adaptation. Understanding these mechanisms will not only shed light on fungal evolution but also provide broader insights into microbial genetics and ecology. It is important to acknowledge that our methods may be biased toward detecting HGT from distant donors, such as bacteria, rather than from closely related fungi. Additionally, the imbalanced sampling of genomes across different fungal lineages could potentially affect the precision of our estimates concerning the number of genes acquired. We believe that with the availability of a greater number of high-quality fungal and other eukaryote genomes through long-read sequencing in the future, additional HGTs will be identified.

## MATERIALS AND METHODS

### The sources of fungi data set used

All publicly available fungi sequences used in this study were obtained from the National Center for Biotechnology Information (NCBI) GenBank database. We focused on Dikarya, a subkingdom of fungi comprising the phyla Ascomycota and Basidiomycota, which represent the majority of known fungal species. All fungi data were downloaded by February 2022, and only one representative genome from every genus of Dikarya was included. Lifestyle-related traits for each genome were annotated using FungalTraits (51), a comprehensive database offering detailed information on fungal characteristics.

### Genome completeness assessment and phylogenetic analysis

Genome completeness was evaluated using BUSCO v5.2.0, which assesses completeness based on the presence/absence of a set of 758 predefined orthologs in the fungi_odb10 database. For each BUSCO gene, its consensus orthologous protein sequence was queried against each genome using tBLASTn, and gene structures were predicted by AUGUSTUS v3.2.2. Predicted genes were aligned to the hidden Markov model profile of the BUSCO gene, with results categorized as “full-length,” “duplicated,” “fragmented,” or “missing” based on alignment characteristics.

To construct the phylogenomic data set, 758 single-copy, full-length BUSCO genes from representatives of Ascomycota and Basidiomycota, along with three out-groups, were utilized. Each gene was aligned with MAFFT (52) v7.490 with default options, and ambiguously aligned regions were trimmed using trimAl (53) version 1.4 with the “gappyout” option. Amino acid alignments with over 50% taxon occupancy were concatenated into the full data matrix. Phylogenetic analyses were conducted using IQ-TREE (54) version 2.0.3 in a maximum likelihood (ML) framework, applying the LG+R5 model, which is widely recognized for its consistent performance in phylogenomic analyses.

### HGT detection

To identify fungal genes potentially acquired horizontally from non-fungal organisms, we employed a robust approach utilizing each gene’s Lineage Probability Index (LPI) score, Alien Index (AI) score, the outgroup lineage (outg_pct), and its placement in a maximum likelihood phylogenetic tree (55–57). The LPI score assesses the likelihood of a gene being acquired through HGT from a foreign organism, rather than through vertical inheritance from a common ancestor, by analyzing the distribution of similar genes in reference genomes from different taxa. The AI score compares gene similarity between specified ingroup and outgroup taxa, while outg_pct represents the percentage of species from the donor lineage in the top hits with different taxonomic species names. In this study, we initially employed similarity-based HGT-detection methods to pre-select candidates from closely related and distant taxa. This approach reduces computational intensity compared to phylogeny-based methods, which require sequence alignments and phylogeny reconstructions (58, 59). Subsequently, candidates underwent verification using a phylogenetic approach.

To reduce spurious results from small genomic fragments of contaminant organisms, we restricted our analysis to genes located in genomic contigs or scaffolds longer than 100 kb, following methods established in previous studies (47). This filtering approach allowed us to focus on 73.9% of protein-coding genes from 829 fungal genomes, totaling 7,091,363 genes out of the 9,597,840 genes identified across these genomes. By excluding shorter contigs, we aimed to enhance the reliability of our HGT detection. We analyzed the impact of varying the threshold percentage of HGT content in contigs on the identification of HGT genes. The thresholds tested were 10%, 20%, 30%, 40%, 50%, 60%, 70%, and 80%. We selected a 50% threshold as a balanced approach, offering a reasonable trade-off between discarding potential contaminants and retaining true HGT genes. Contigs comprising ≥50% HGTs were discarded as contamination (44). Variable contamination levels were observed in some genomes, notably in *Neocamarosporium betae* and *Hygrocybe conica* genomes (Table S8).

For each gene, we evaluated horizontal acquisition using a two-step workflow. Initially, we conducted a BLASTP search against the NCBI non-redundant protein database (last accessed 20 January 2022), employing an e-value cutoff of 1. DarkHorse (55) pre-screened candidate genes in fungal genomes to identify potential donors and recipients of transferred genes, with a filter threshold set at 10% (LPI score). This process identified 29,796 genes as candidate HGTs.

Subsequently, AvP (56) verified these candidates with an AI value >0 and outg_pct >80%, calculating the AI (57) and the percentage of species from the outg_pct among the top 200 hits with different taxonomic species names. The 200 most similar homologs were aligned using MAFFT and trimmed for ambiguously aligned regions with trimAl. ML trees were inferred using IQ-TREE, employing the best-fit model of amino acid evolution as determined by ModelFinder (60), with 1,000 ultrafast bootstrap replicates to assess branch support. Each phylogenetic tree was processed to classify query sequences as HGT candidates, ensuring a comprehensive and accurate approach to HGT detection. Only genes with HGT or HGT-NT tags were considered putative HGT-acquired genes, resulting in the identification of 20,093 HGT-acquired genes. HGT-NT tags refer to horizontally transferred genes where no closely related species (ingroup) were identified, indicating an absence of closely related fungal species for that specific transfer (56). The phylogenies of these HGT-acquired genes indicate they stem from 8,815 distinct HGT events: 6,953 were species-specific, and 1,862 involved two or more species (Table S2).

### Validation of HGT-acquired genes

To validate the presence of HGTs across different fungal species, a subset of genes identified as acquired through HGT were randomly selected for validation via PacBio/Illumina sequencing. Specifically, four distinct fungi species (*Gymnopilus junonius, Hypholoma sublateritium, Galerina marginata,* and *Flammulina velutipes*) harboring HGT-acquired genes were subjected to sequencing. Basidiospores were cultivated on potato dextrose agar (PDA) and incubated at 25°C until spore germination occurred. Subsequently, single spore isolates devoid of clamp connections were selected and individually sub-cultured on PDA plates at the same temperature. High-quality genomic DNA extraction was performed using the QIAGEN Genomic kit (Qiagen, Dusseldorf, Germany) following standard protocols. DNA integrity was assessed via agarose gel electrophoresis, and quantification was carried out using the Qubit 2.0 Fluorometer (Thermo Scientific).

For *Gymnopilus junonius*, *Hypholoma sublateritium*, and *Galerina marginata*, single-molecule real-time (SMRT) sequencing libraries were constructed with an insert size of 20 kb using the SMRT bell TM Template kit, version 1.0. Whole-genome sequencing was conducted on the PacBio Sequel platform at Beijing Novogene Bioinformatics Technology Co., Ltd. For *Flammulina velutipes,* whole-genome sequencing was performed on the MGISEQ-2000 platform at BGI (Shenzhen, China), with library insert sizes of 350 bp and 150 bp pair-end sequencing. Validation criteria were set such that an HGT-acquired gene would be deemed validated if its amino acid sequence exhibited a similarity of over 70% to sequences present in our analyzed data.

In addition to genomic validation, transcriptome data were retrieved to assess the expression levels of the identified HGT-acquired genes. Thirteen transcriptome data sets corresponding to 13 fungi species were obtained from the NCBI Sequence Read Archive Browser (https://www.ncbi.nlm.nih.gov/sra). The raw reads were aligned to the corresponding reference genomes using HISAT2 (61) with default parameters. Gene expression levels for each HGT-acquired gene were quantified using the Fragments Per Kilobase of exon model per Million mapped fragments metric, with transcript assembly and quantification performed using StringTie (62).

### Genome assembly and gene annotation

For PacBio reads, genome assembly was performed using flye (63) version 2.9-b1768, a long-read metagenomic assembler that constructs a repeat graph to assemble and polish contigs. Illumina Reads were assembled using Platanus-allee (64) version v2.2.2. Gene prediction was analyzed using GeneMark-ES version 4.71_lic (65), which has a special option for fungal genomes to account for fungal-specific intron organization. Virulence factors were predicted using the VFDB (VFDB_setA_pro.fas) (66) and PHI (67), with a threshold for amino acid similarity set at >70%. Secretory proteins were identified using SignalP 6.0 (68). GO terms were retrieved using InterProScan v5.53-87.0 (69), and their distribution across categories was analyzed and visualized using WEGO2 (70). GO enrichment analyses were performed using clusterProfiler (71). In this analysis, the specific HGT-acquired gene sets were compared against the background of all HGT-acquired genes. Ka/Ks values were calculated using KaKs_calculator 3.0 with the NG model for all pairs of HGT genes (72). Amino acid alignments for gene pairs were initially generated using MAFFT, followed by conversion of the results into coding sequence alignment using PAL2NAL (73). These alignments were then processed through KaKs_Calculator to derive Ka/Ks values. Transposable elements were annotated using ISfinder (74), and the distance of each gene to the nearest transposable element was calculated and compared. Statistical significance was calculated using the Wilcoxon rank sum test.

### Statistical analysis

RDA was conducted using the vegan package in R to evaluate the relationship between lifestyle-related traits and HGT event diversity across fungal species. We employed 1,000 permutations to ensure the robustness of the RDA results. To account for phylogenetic effects, PGLS regressions were performed using the gls function from the nlme package, allowing us to assess whether lifestyle-related traits were significant predictors of HGT diversity while controlling for species' phylogenetic relatedness. Graphs were drawn and analyzed using R 4.0.3 [38]. A significance level of *P*-value < 0.05 was considered statistically significant.

## Data Availability

All the genomic sequence data sets are available in the NCBI GenBank under accession PRJNA1134811.

## SUPPLEMENTAL MATERIAL

Supplemental Figures mbio.02855-24-s0001.pdf Figures S1 to S9.

Legends mbio.02855-24-s0002.txt Legends for Figures S1 to S9.

Supplemental Tables mbio.02855-24-s0003.xlsx Tables S1 to S8.

ASM does not own the copyrights to Supplemental Material that may be linked to, or accessed through, an article. The authors have granted ASM a non-exclusive, world-wide license to publish the Supplemental Material files. Please contact the corresponding author directly for reuse.
