# Supplementary figures and images for: Uneven distribution of prokaryote-derived horizontal gene transfer in fungi: a lifestyle-dependent phenomenon
Source: mBio. 2024 Nov 29;16(1):e02855-24. doi: 10.1128/mbio.02855-24 (PMC11708051; doi:10.1128/mbio.02855-24)

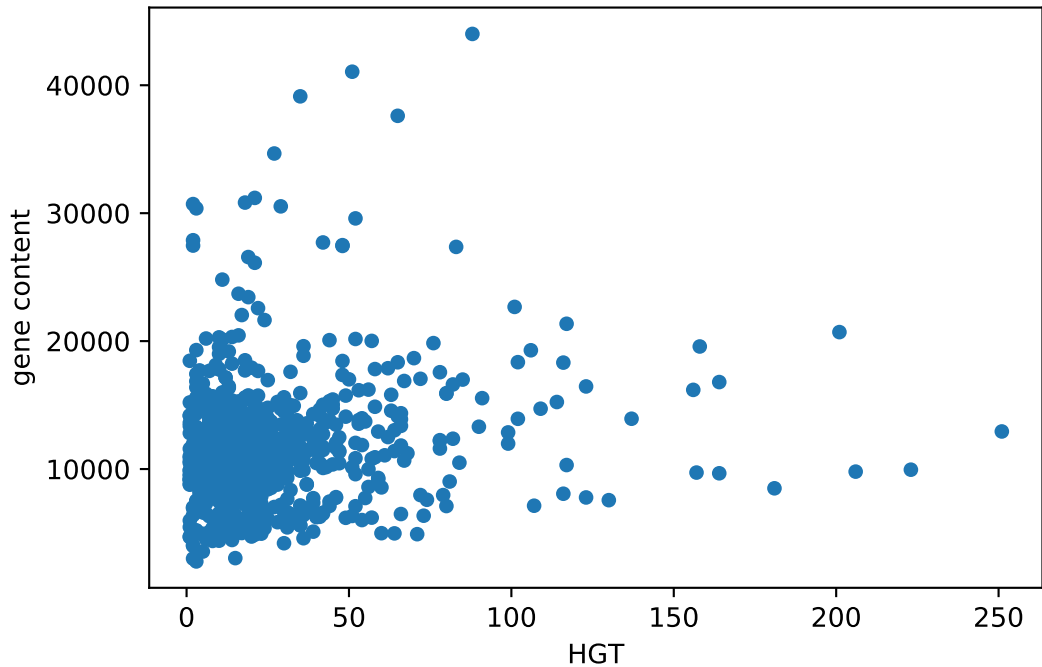

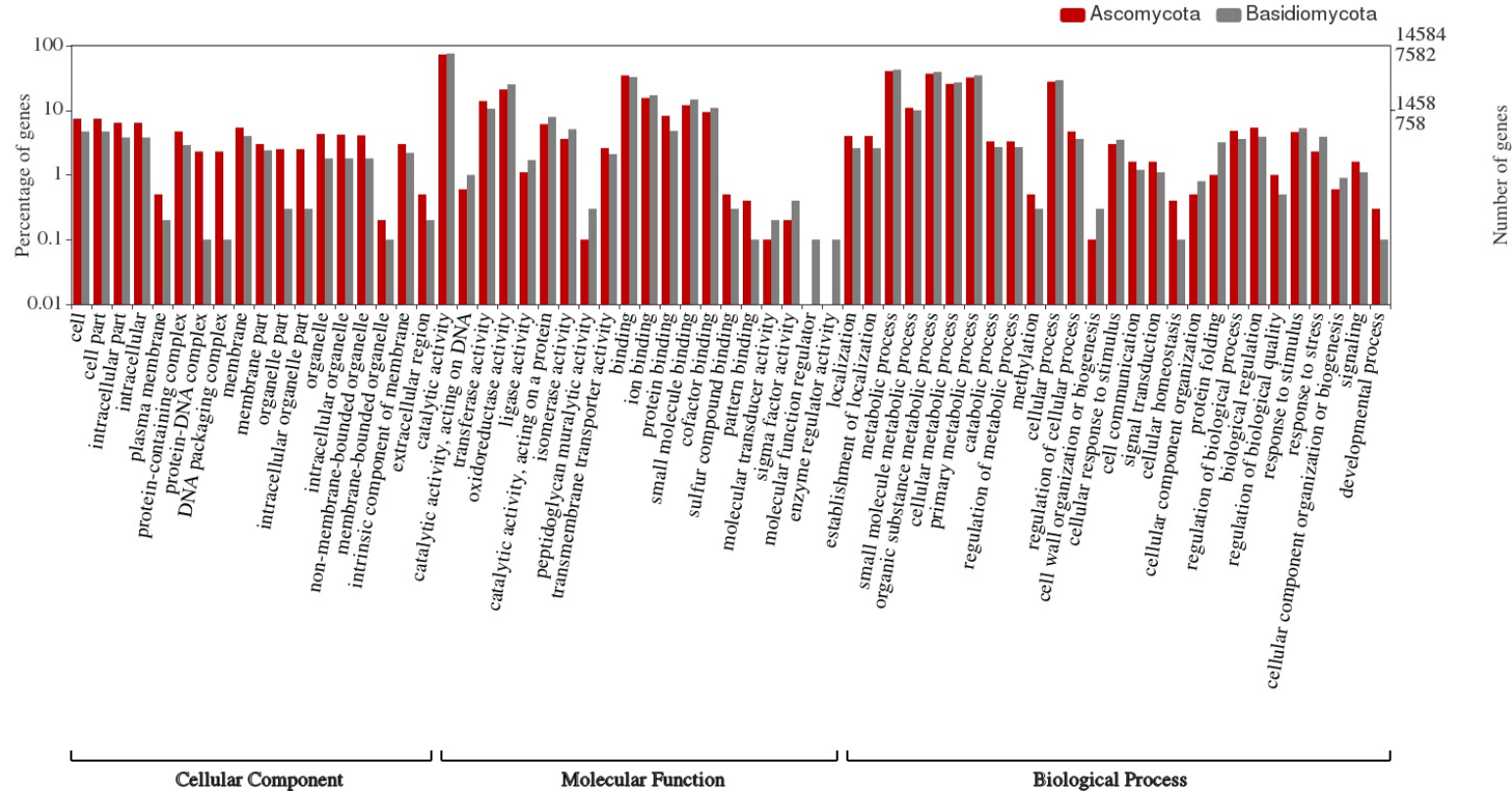

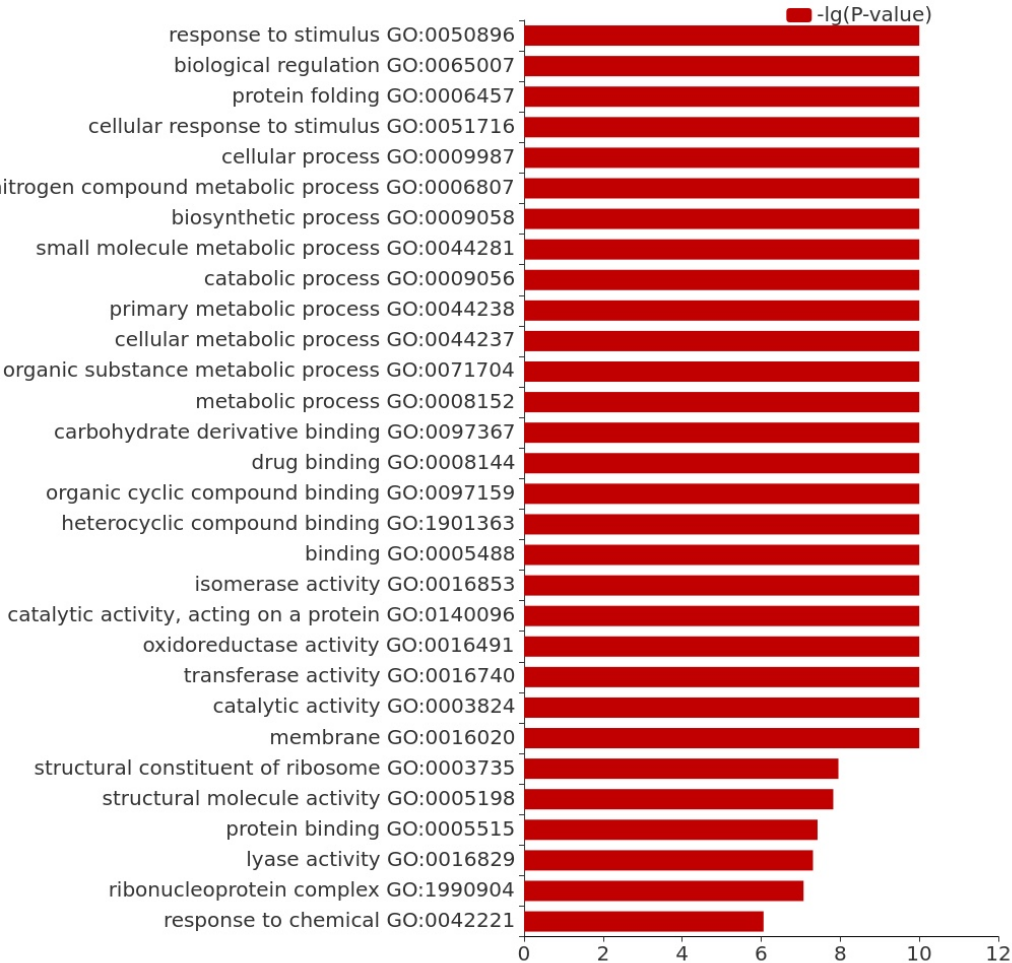

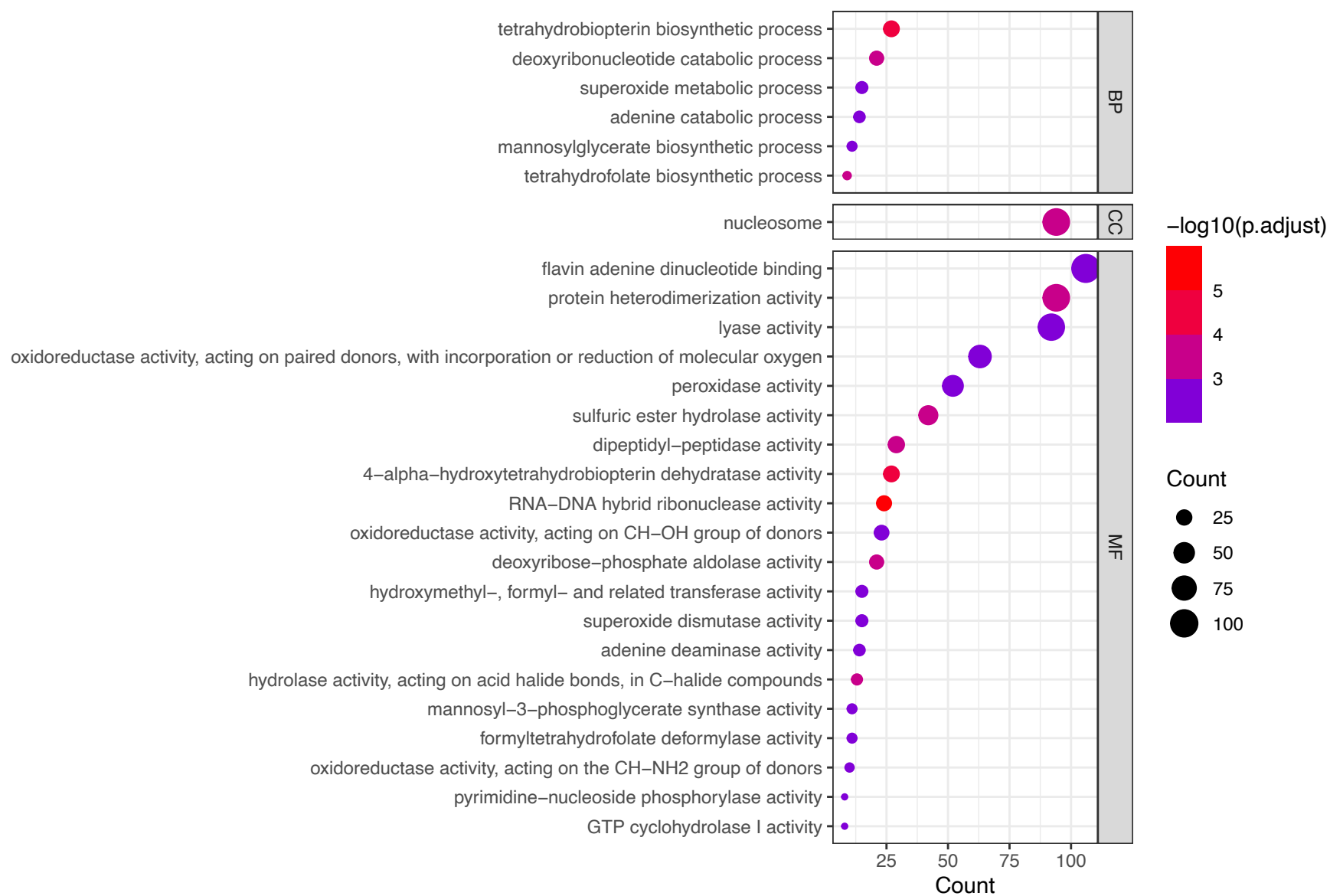

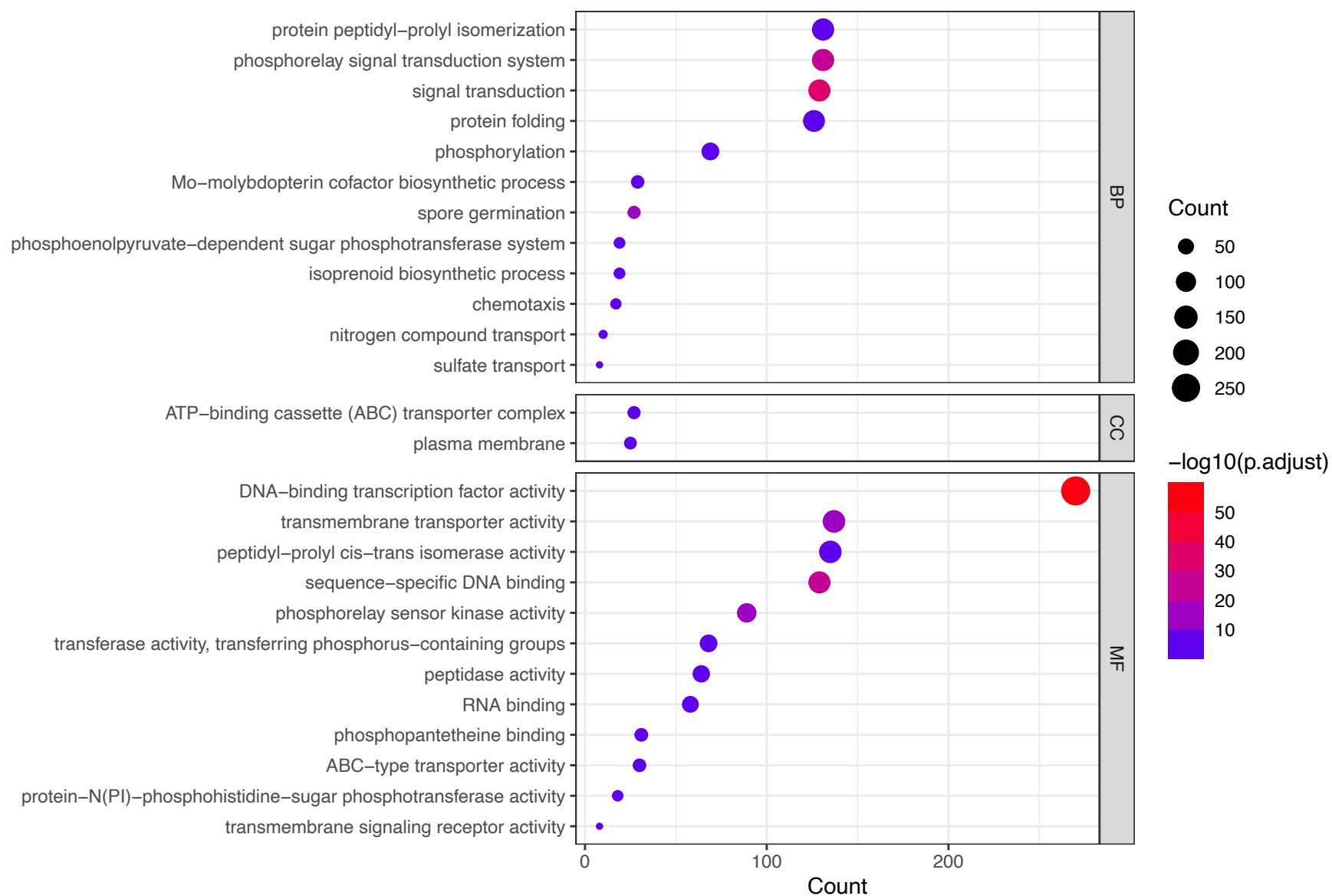

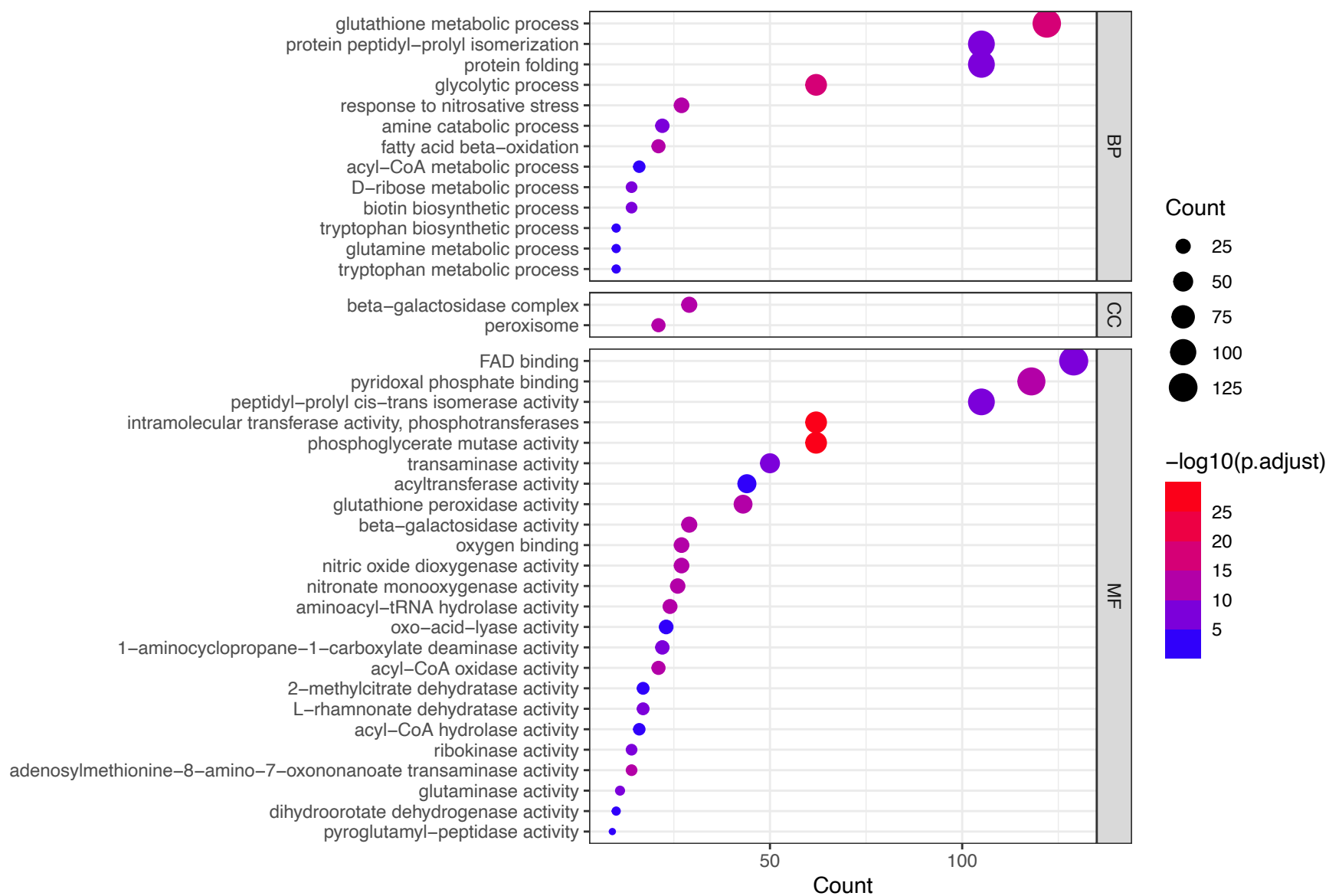

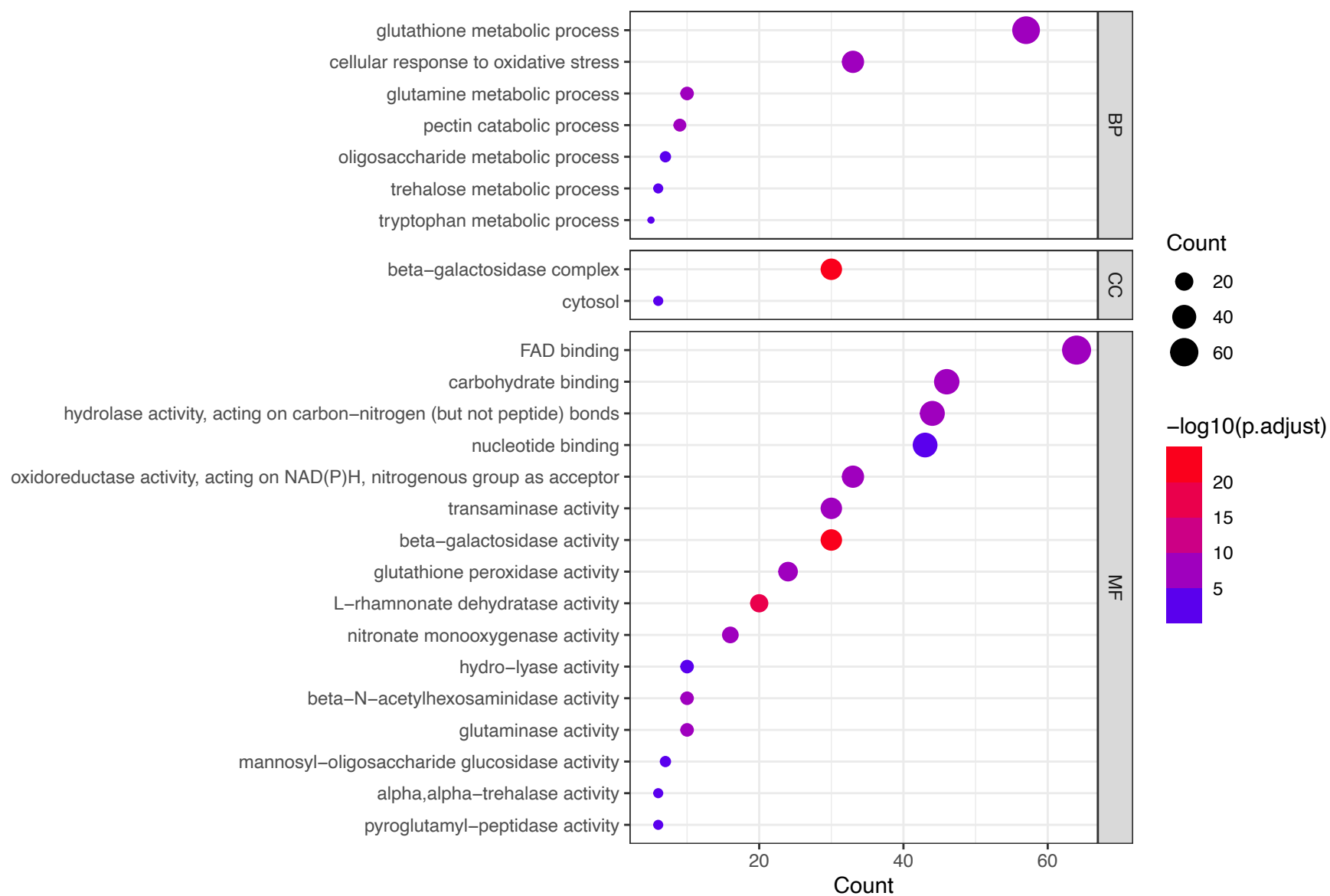

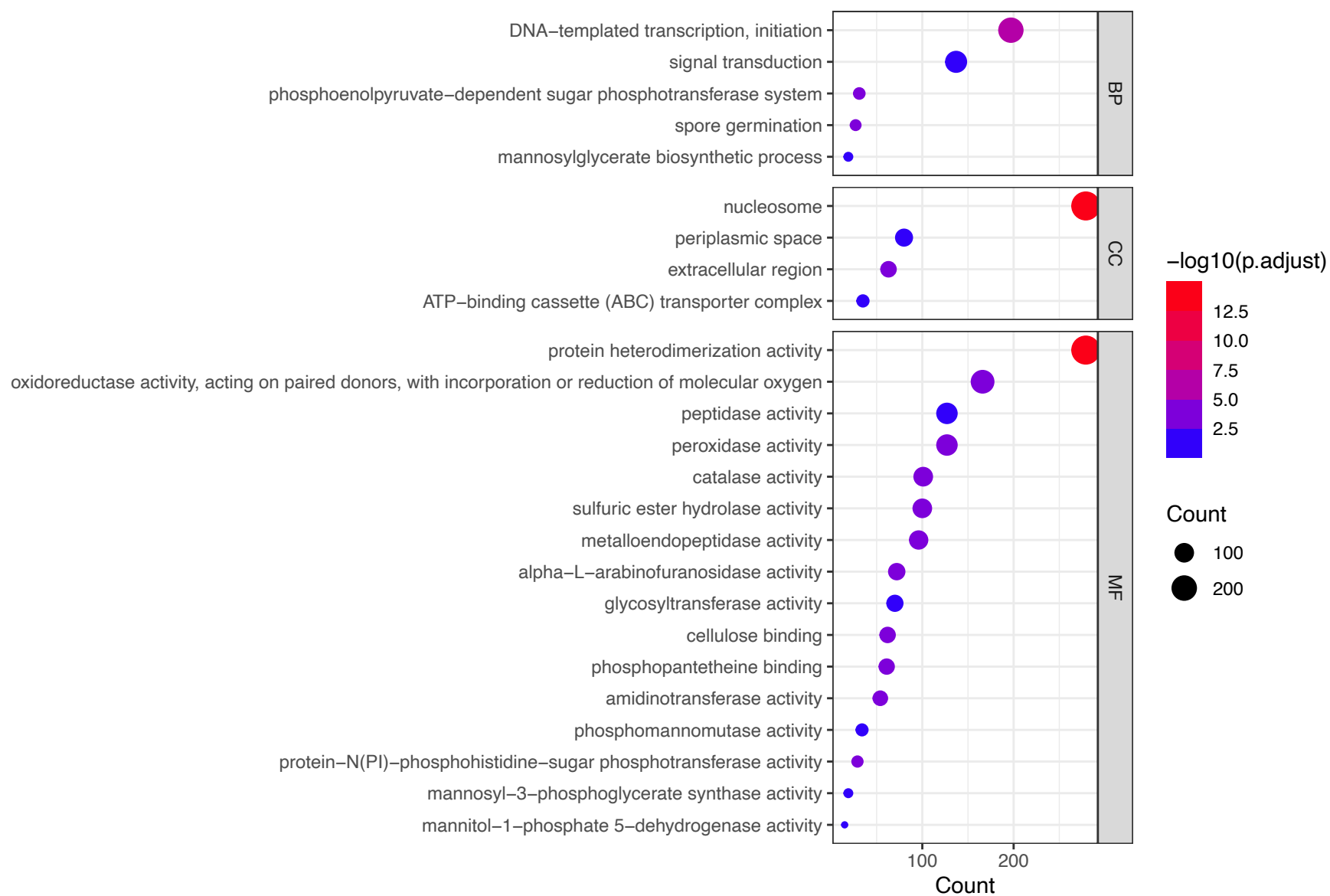

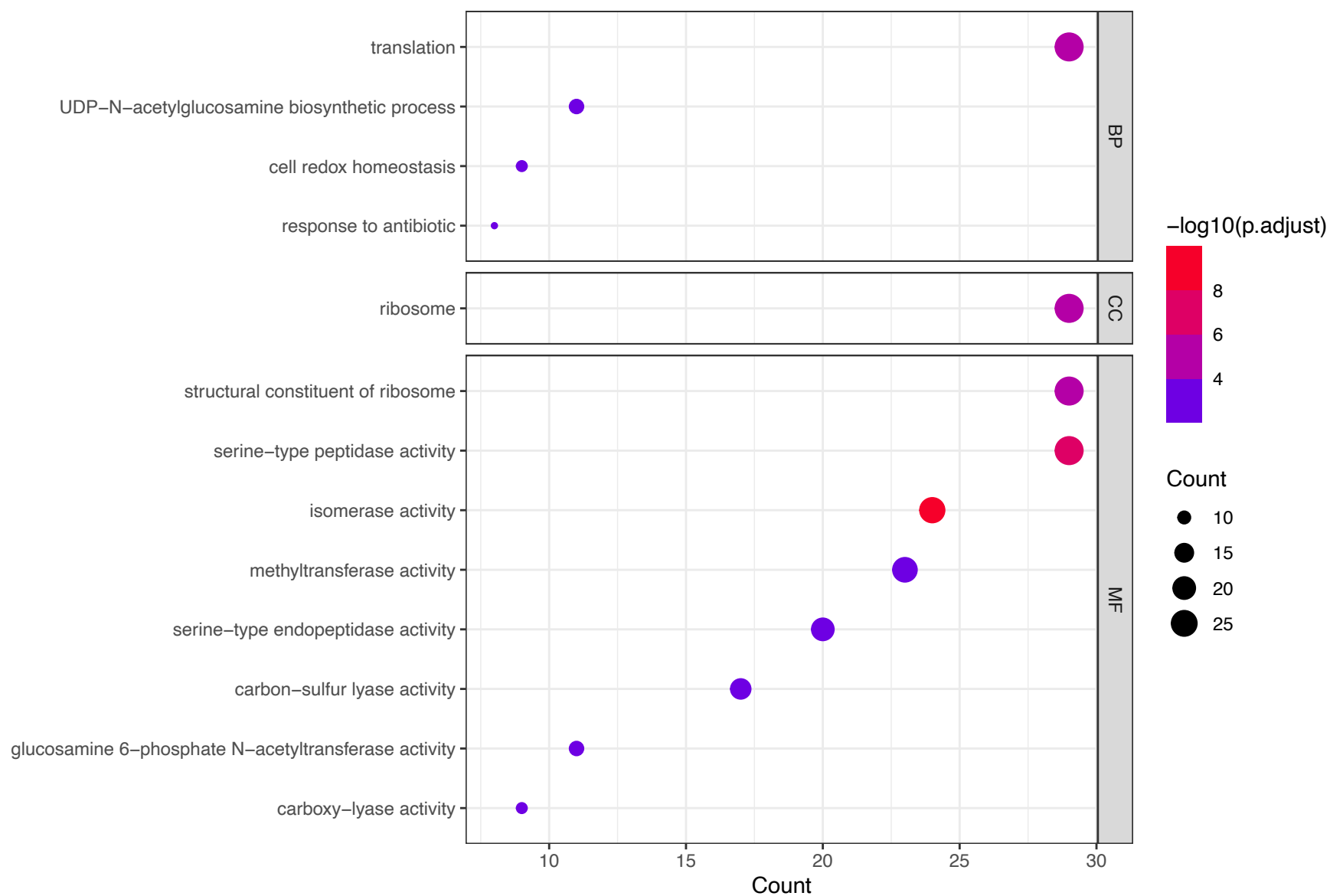

Supplement: Supplemental Figures — Figures S1 to S9. [file mbio.02855-24-s0001.pdf]
